# Structural design of memory system for Ternary Optical Computer

**DOI:** 10.1371/journal.pone.0309839

**Published:** 2025-02-28

**Authors:** Honghong Zhang, Yi Jin, Hongjian Wang, Xunlei Chen, Zhehe Wang, Shan Ouyang, Shuang Li

**Affiliations:** 1 School of Information Engineering, Henan University of Animal Husbandry and Economy, Zhengzhou, China; 2 School of Computer Engineering and Science, Shanghai University, Shanghai, China; 3 School of Computer Science and Technology, Donghua University, Shanghai, China; 4 College of Information, Mechanical and Electrical Engineering, Shanghai Normal University, Shanghai, China; Siksha O Anusandhan University School of Pharmaceutical Sciences, INDIA

## Abstract

Ternary Optical Computer (TOC) is unique in the development of optical computers, in terms of principle, experiment, algorithm and application. After 20 years of development, six generations of prototypes have been developed. At present, tri-state optical signal storage is the main problem faced by TOC. According to the characteristics of tri-state optical signals and the ternary optical processor’s special requirements for storage systems, we design and implement the interface structure of TOC memory system, including the overall structure of the interface, the address generation module for memory access, the data input and output channels, the read/write timing, and the working process of the memory interface. Finally, the correctness of the memory system interface design is verified by the experiments, which are carried out on FPGA, of reading and writing operational result data of the SD16 TOC prototype. This work tries to improve the theoretical system and practical basis of TOC.

## 1. Introduction

The TOC is an optoelectronic hybrid computer with optical operation and electric control, which is one of the important members of various new computers. In recent years, TOC has made great progress. Programming model [1], computing-data file [2], address calibration of decoders [3] and other technologies have been born. Discrete Fourier transform [4], cellular automata [5], walsh-hadamard transform [6], wavelet transform [7], parallel artificial fish schooling algorithm [8], parallel genetic algorithm [9] and other algorithms that can utilize the advantages of TOC computing have been emerging. Recently, Jin et al. [10] discussed the related theory, principle and structure of the ternary optical logic operator from the latest TOC prototype SD16. The TOC expresses ternary information by adopting no-light state, horizontally polarized light state and vertically polarized light state, where it completes computing by changing the light state. Therefore, the input data and operation results of TOC are expressed by three kinds of optical signals: no-light, horizontally polarized light and vertically polarized light, and TOC memory system needs to solve the problem of storing the ternary information carried by tri-state optical signals.

In recent years, multi-wavelength multi-order optical storage technology [11], dual-beam super-resolution optical storage technology [12], holographic storage technology [13], glass storage technology [14], near-field optical storage technology [15] and other new optical storage technologies have been emerging. Most of these technologies utilize some physical or material properties caused by the optical state to store the information carried by the optical signal, and these technologies are mainly used to store binary data. At present, one of the state-of-the-art technologies for directly storing optical state was proposed by Ma et al. from the University of Science and Technology of China in 2021 [16], when they used ^151^Eu^3+^:Y_2_SiO_5_ crystal to store optical signals for an hour. Before that, a team from the University of Darmstadt in Germany used Pr^3+^:Y_2_SiO_5_ crystal to store light for one minute [17]. However, this technology is still in the experimental stage.

Limited by the scarcity of optical state memory devices, the TOC currently uses electronic storage devices with mature technology and low cost. The widely used semiconductor memories can only store binary electrical signals in bytes, with a maximum data bus width of 8 bytes. The I/O data of Ternary Optical Processor (TOP) are tri-state optical signals, and the TOP can handle a large number of trits in parallel. For example, a single module of SD16 has 192 processor trits. The corresponding ternary data bus width is 192 trits. If two bits are used to express one trit, the corresponding binary data bus width of I/O data is 384-bit for a single module of SD16. For memory, TOP is its most important input and output device, and the signal difference between TOP and memory puts new requirements on the memory interface. Designing I/O interface components suitable for the combination of TOP and semiconductor memory to ensure correct and fast access to information has become an urgent problem to be solved.

In this paper, we take SD16 as the research background and experimental platform, where we design the structure of TOC memory system for solving the problem of storing the information carried by the many trits of tri-state optical signals in TOC. Our work includes the overall structure of the memory system interface, the structural design of the address generation module for memory access, the design of data input and output channels, the read/write timing of the memory interface and its working process, which all together realize the conversion and storage of TOP’s I/O data. Our work verifies the feasibility and effectiveness of the structural design of memory I/O interface, thus promoting the TOC towards industrialization.

## 2. Design philosophy and overall structure of the memory system interface

### A. Design philosophy of the memory system interface

The features of TOP include parallel optical operation, numerous data trits, trit-by-trit grouping, trit-by-trit reconfigurability, etc. These features impose new requirements on the memory interface and give rise to our design concept. The design ideas for the functionality of TOC memory system can be summarized as four parts: First, the form of physical signals expressing data is converted between tri-state optical signals and binary electrical signals. The input signals and the output result signals of TOP are the tri-state optical (no-light, horizontally or vertically polarized light) signals, whereas the binary electrical signals are stored in memory. So the interface has to smoothly convert the expression of information between tri-state optical signals and binary electrical signals without any noticeable delay, while ensuring the information remains unchanged Second, the buffering and latching of a large amount of data. Parallel optical operations of TOP have many input/output data trits, and the interface has to cache many data for next transmission. Third, to design a proper transmission method to solve the problem of limited width of data transmission channel in the current memory system. Fourth, it is necessary to provide communication signals between TOP and memory. The above features inevitably impose corresponding new requirements on memory addressing.

Both the input and output of TOP are ternary data, i.e., there are three desirable symbols for each data. In order to be compatible with the binary data of electronic computers, researchers have adopted the encoding of two binary symbols to represent a ternary trit. Two binary symbols have four codes namely 00, 01, 10 and 11. In principle, one can choose any three of these codes to indicate a ternary trit: the specific choice depends on the convenience of solving the problem or facilitating the production of devices. In order to make the device or software with different coding choices to exchange information correctly, researchers agreed on a basic coding rule: 00 represents ternary trit 0, 01 represents ternary trit 1, 10 represents the third ternary trit u, and 11 represents special information [18]. The external data of any software or device with binary encoding are to comply with this agreement. Given that memory is the basic device of a computer system, basic encoding is used to express ternary data in all the memories described in this paper.

### B. Overall structure of memory system interface

**Fig 1:**
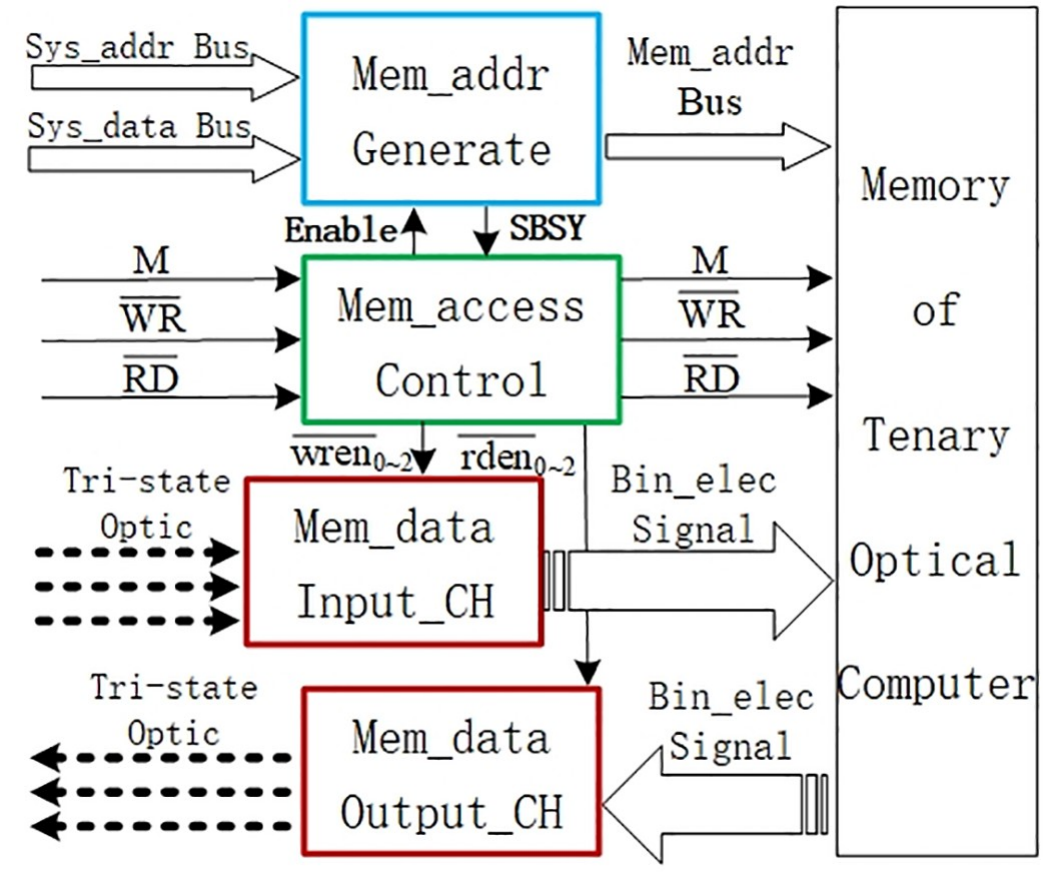
Overall structure of memory system interface.

According to the above design philosophy of the memory system interface, we design the overall structure of the interface and its components as shown in Fig 1. In accordance with the different functions, the interface is divided into four modules, which are labeled by the blue, green, and upper and lower two red boxes in the middle of Fig 1. The memory address generation module in the blue box receives the address signals sent by the system through the address and data buses, and this module generates memory access address. The memory read/write control module in the green box receives read/write control signals from the system and coordinates the modules to work together for accessing TOC memory. The memory data input channel module in the upper red box is used to receive the tri-state optical signals of TOP operation results, converting them into corresponding binary electrical signals. This module then writes the binary data into the TOC memory according to the memory address indicated by the address bus under the control signal. The memory data output channel module in the lower red box has the opposite function to the input channel module: it receives binary electrical signals from the TOC memory, which are converted to tri-state optical signals by an optical signal generator inside the module and then sent to the TOP for operation.

The two hollow rightward arrows on the left side of the blue box in Fig 1 represent the system address bus and data bus respectively, while the hollow rightward arrow on the right side represents the memory address bus. The black solid arrows around the green box indicate control or status signals. When the M signal is high, the memory is to be accessed, where $\bar{WR}$ corresponds to writing signal and $\bar{RD}$ corresponds to reading signal. The Enable arrow represents a control signal, controlling the start of the address generation module; and the SBSY (State BuSY) arrow indicates whether the state of address generation module is busy. The ${\bar{wren}}_{0\sim 2}$ arrow represents three write control signals, controlling the start of three submodules of the data input channel. The ${\bar{rden}}_{0\sim 2}$ arrow represents three read control signals, controlling the start of three submodules of the data output channel. The dotted lines with arrows on the left side of red boxes indicate tri-state optical signals, with the rightward arrows connecting to the input channel and the leftward arrows connecting to the output channel; the dotted-tailed arrows on the right side of red boxes indicate the binary electrical signal buses, where the input and output channel modules are connected in opposite directions. The rightmost box in Fig 1 represents the TOC memory, which currently uses the memory device of electronic computer.

## 3. Design of each functional module of memory interface

### A. Architectural design of the memory address generation module

**Fig 2:**
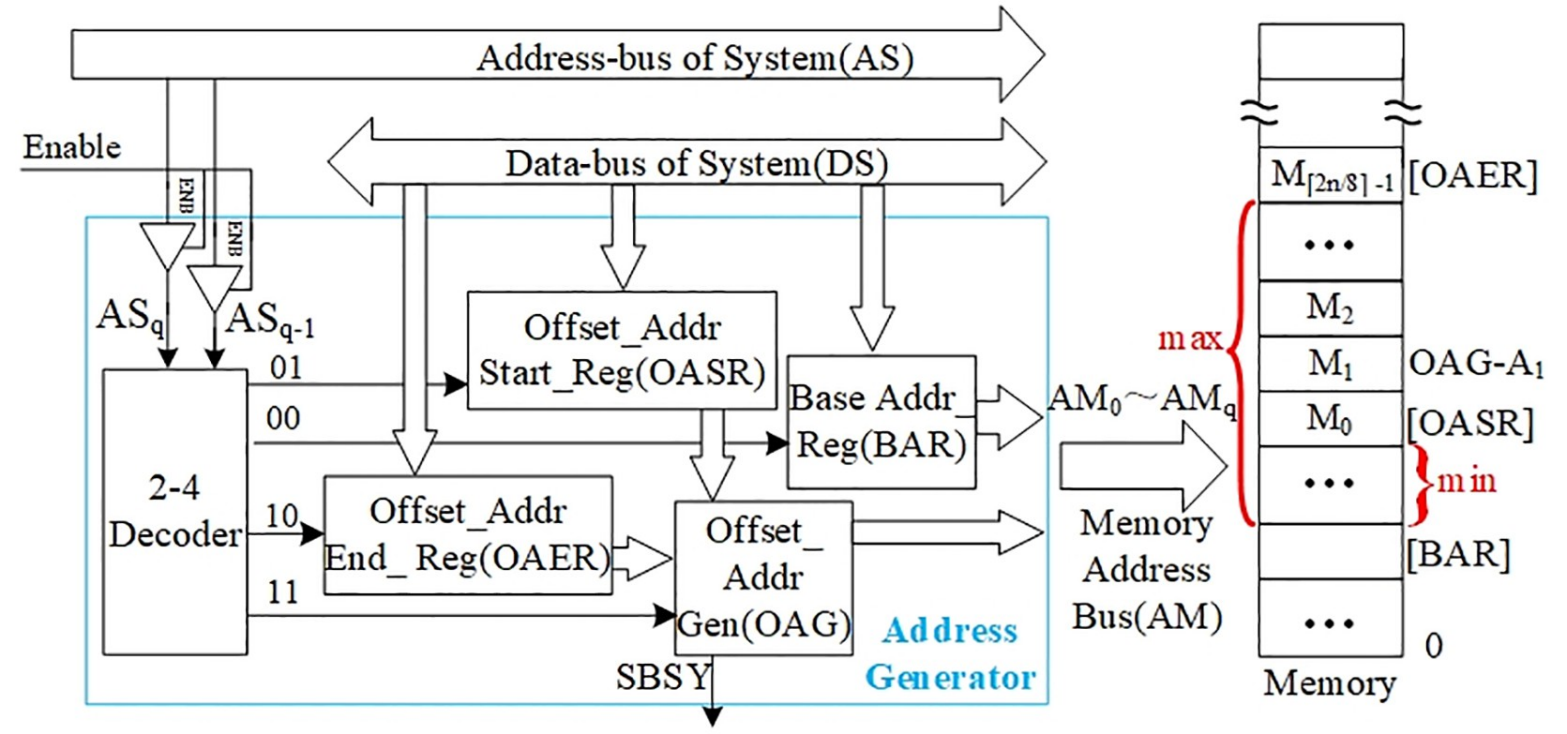
Architecture of memory address generation module.

The structural design of the memory address generation module is shown in Fig 2. The blue box is the address generation module, and it is connected to the system address bus AS (Address of System), the data bus DS (Data-bus of System), and the memory address bus AM (Address of Memory).

In view of the fact that the many trits and the reconfigurability of the operation rules of the TOP make it more efficient in processing large amounts of data, the TOP often has to compute many sets of data for a single task. To suit this feature, we set a Base Address Register (BAR), an Offset Address Start Register (OASR), and an Offset Address End Register (OAER) in the address generation module. The BAR is used to indicate the first address of the memory block for all the data of the task. The OASR indicates the offset of the smallest address of the memory block of the data involved in the current operation, from the first address indicated by BAR. The OAER indicates the offset of the largest address of the memory block, from the first address indicated by BAR. The address relationships expressed by these three registers are labeled in the rightmost memory part of Fig 2 with [BAR], [OASR], and [OAER]. In Fig 2, *max* and *min* indicate the maximum and minimum distance of the memory interval from the first address, respectively. These three register values are written by the control processor via the system buses AS and DS. An Offset Address Generator (OAG) is also set up in the address generation module to generate the current offset address and to guarantee that the address of memory cell actually accessed does not cross the memory interval bounded by OASR and OAER. The memory cell M0 in Fig 2 indicates the first cell of the memory interval, i.e., the memory cell labeled with [OASR]. The memory cell M1 indicates the second cell of the memory interval, i.e., the memory cell labeled with OAG-A_1_ corresponding to the next address generated by the OAG with respect to [OASR].

AS_q_ and AS_q-1_ in the system address bus address the four address registers contained in the Address Generation Module through 2–4 decoder, and the addressing codes are listed in Table 1.

**Table 1:** Addressing table for registers within address generation module.

AS_q_, AS_q-1_	00	01	10	11
addressed register	BAR	OASR	OAER	OAG

When BAR, OASR and OAER are addressed, its data input receives the address preset value fed by DS, and when not addressed, it saves and outputs the current preset address value. When OAG is addressed, it first outputs 1 from the status signal SBSY and holds it, representing the beginning and in progress of TOP’s access to the memory. At the same time OAG initializes its own accumulator with the value of OASR and generates the first current offset address. At the end of each access, the accumulator generates the next offset address and compares it with the value of OAER, and when it is not out of bounds, it starts the process of a new access until the newborn offset address is out of bounds. At this moment the SBSY of OAG outputs 0, representing the end of this TOP access memory.

The output signal buses of BAR and OAG are combined to connect to the address buses of memory, noted as AM_0_~AM_q_.

### B. Structural design of memory data input channel

**Fig 3:**
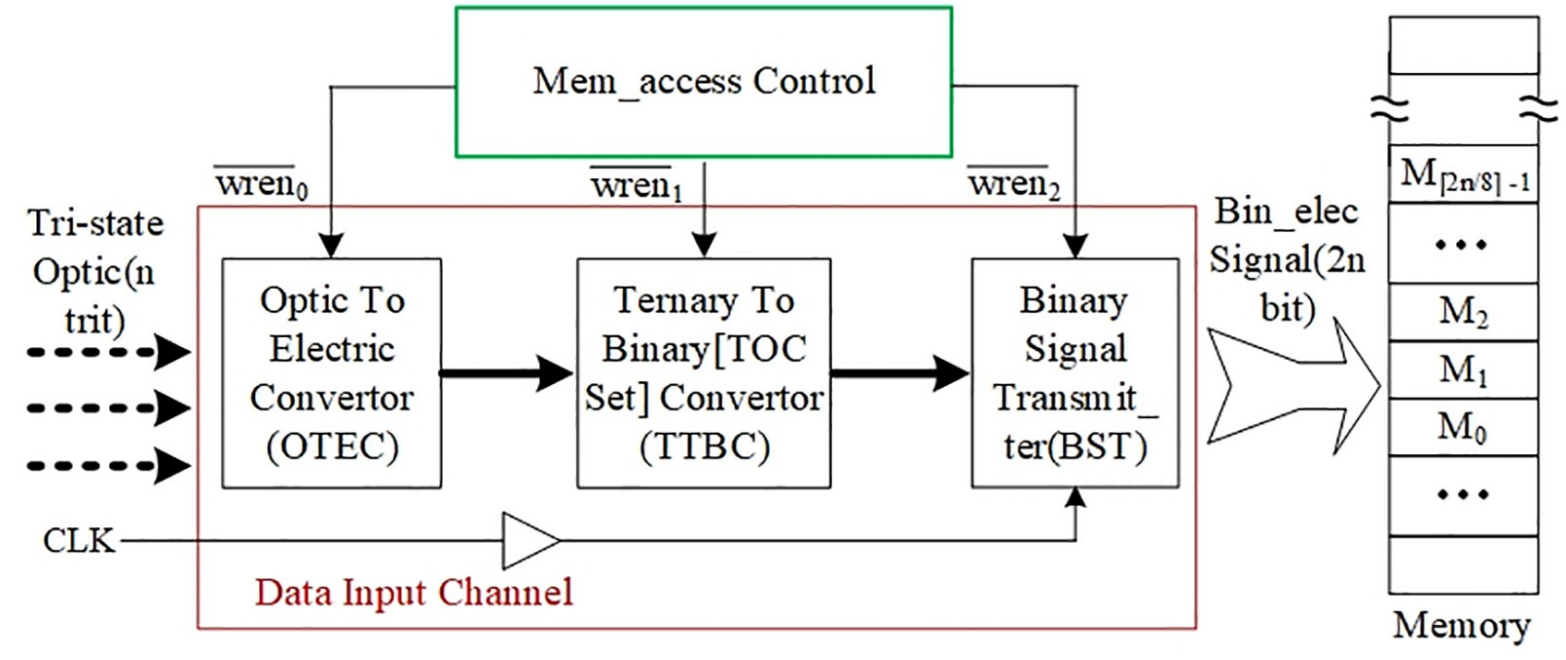
Structure of the memory data input channel.

The correspondence between the ternary data and the three optical states may be different in different TOP structures, and thus, before the output of a TOP is saved to the memory, each tri-state optical signal is firstly converted into a corresponding tri-state electrical signal, and then each tri-state electrical signal is converted into two-bit code of two-state electrical signals, which (often referred to as two-bit binary electrical signals) have electrical characteristics suitable for the physical requirements of the memory. After completing these two steps of conversion, the generated binary electrical signals can be directly written to the memory, and each two-bit code represents one-trit of ternary data, which can be directly used by any ternary software or ternary device, thus realizing the preservation of ternary data. Based on this idea, we design the structure of the memory input data channel as shown in Fig 3.

In the data input channel of Fig 3, the left functional module is the optoelectronic signal converter OTEC (Opitic To Electric Converter), which serves to convert the n-trit tri-state optical signals of the TOP operation result into the corresponding n-trit tri-state electrical signals under the control of signal ${\bar{wren}}_{0}$. The core hardware of this module includes the optoelectronic converter element and the light polarization direction recognizer element. The functional module in the middle is the Ternary To Binary Converter (TTBC), which converts each tri-state electrical signal into the corresponding two-bit binary electrical signals under the control of signal ${\bar{wren}}_{1}$. The functional module on the right is the Binary Signal Transmitter (BST), which performs a more detailed conversion of the electrical signals under the control of signal ${\bar{wren}}_{2}$. It has an input data width of 2n-bit, an output data width of 64-bit, and several internal 2n-bit registers to buffer the input data.

Each 2n-bit data is divided into 2n/64 64-bit data, and then these 64-bit data are continuously output to the memory, and each 64-bit data are written to the memory under the control of the clock signal CLK. Since the 2n-bit data are divided into a number of 64-bit data groups to be continuously fed to the memory, the scalable arrow on the right side of the data input channel is labeled "2n-bit electrical signal".

On the rightmost side of Fig 3 is the memory, assuming that the 2n-bit data to be saved are stored starting from the M_0_ cell. In order to be consistent with most existing electronic memories, we setting each cell to store 8-bit data. So a minimum of ⌈2n/8⌉ memory addresses are required, i.e., the last memory cell is M_⌈2n/8⌉-1_.

#### 1. Principle of OTEC

**Fig 4:**
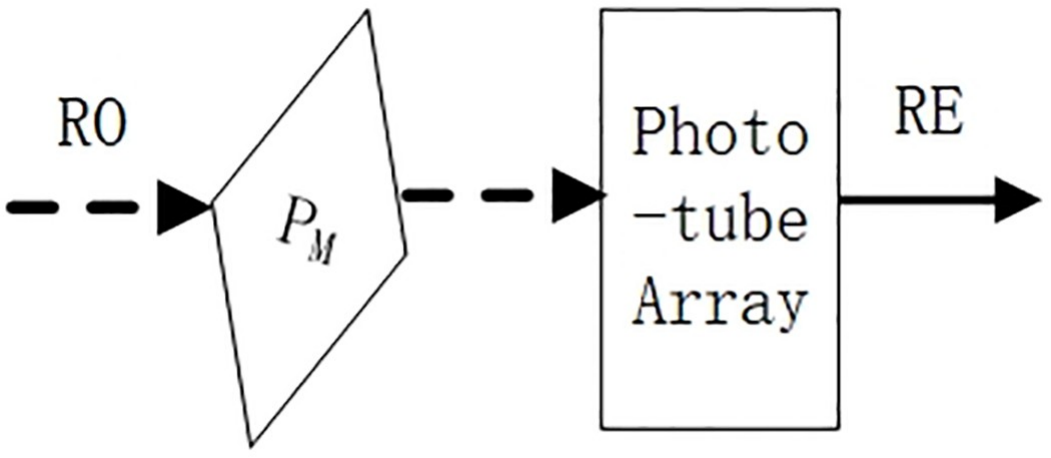
Schematic structure of optoelectronic signal.

The schematic structure of the optoelectronic signal converter OTEC is shown in Fig 4.

In Fig 4, RO (Result Optics) is the result of the tri-state optical signal output from the optical operator, represented by a dotted line. RO goes through the Phototube Array and is converted to an electrical signal result RE (Result Electronics), represented by a solid line. Phototube is an energy integration device, where the size of its output electrical signal is approximately proportional to the intensity of the light it receives. However, the Phototube can not directly determine the polarization direction of the optical signal.

To solve this problem, we apply Marius’ law [19]. The intensity of a line polarized light with an intensity of I_0_, transmitted through a polarizer (without taking absorption into account) is I = I_0_ cos^2^θ, where θ is the angle between the direction of light vibration of the incident line polarized light and the direction of polarization of the polarizer. Taking θ to be 30°, the transmitted light intensity of the vertically polarized light is I_v_, and the transmitted light intensity of the horizontally polarized light is I_h_. Then, I_v_ = I_0_ cos^2^30° = 3I_0_ /4, I_h_ = I_0_ cos^2^60° = I_0_ /4. From the calculations, it is known that the difference in the intensity of the vertically polarized light and the horizontally polarized light is two times when the polarizer P_M_ placed in Fig 4 is tilted by 30°. When these two light intensities are irradiated into the photoelectric tube, the intensity of the output signal differs by a factor of 2. Considering the fact that the intensity of the output signal is 0 when there is no light input into the photoelectric tube, it is clear that the structure given in Fig 4 is capable of determining a tri-state optical signal. We call it a tri-state photoelectric signal converter.

#### 2. Principle of TTBC

**Fig 5:**
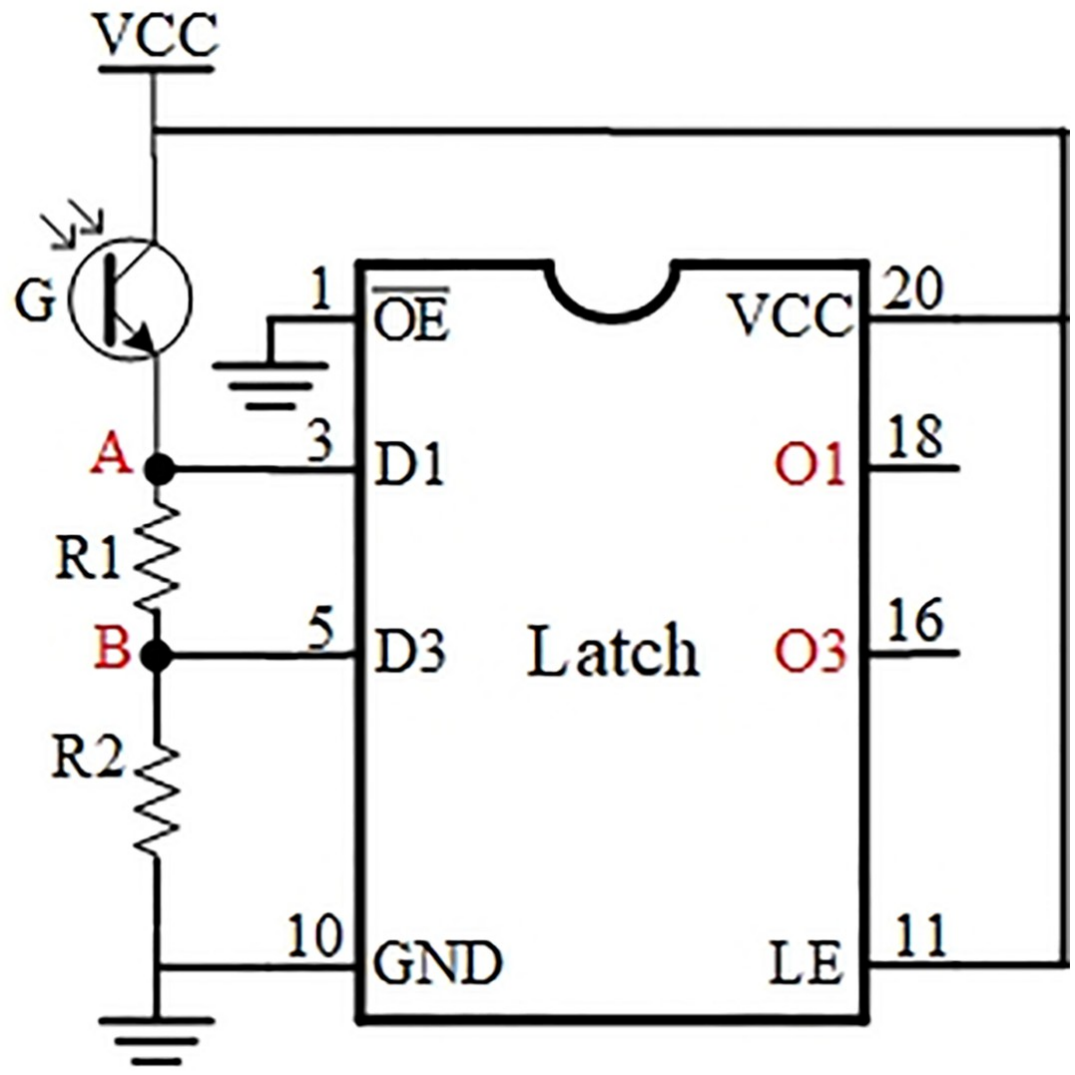
Principle of TTBC (one bit).

The tri-state optical signals output from TOP are converted to tri-state electrical signals after passing through the optoelectronic converter. In order to parse out the correct operation result and store it in the semiconductor memory, it is necessary to convert the electrical signal from ternary to binary. This work is done by the TTBC as shown in Fig 5.

The component G on the left side of Fig 5 indicates a tri-state photoelectric signal converter, which receives tri-state optical signal expressing the result of the operation output from TOP, and outputs high, medium, and low voltage values, i.e., one-trit ternary electrical signal. Then the voltage divider circuit composed of G and two resistors R1 and R2 converts one ternary electrical signal into two binary electrical signals. Points A and B of this voltage divider circuit are connected to a latch’s data input ports (e.g., D1 and D3 in Fig 5), and the latch locks the result of the voltage divider and outputs it through the output ports (e.g., O1 and O3 marked in red in Fig 5).

The resistance value of a three-state photoelectric signal converter G is inversely proportional to the intensity of the light it receives: the greater the light intensity, the smaller the resistance value. When G receives vertically polarized light with the maximum light intensity, the resistance value is minimum, denoted as RGmin; when G receives a light state with the minimum light intensity (i.e. no-light state), the resistance value is maximum, denoted as RGmax; when G receives horizontally polarized light with the middle intensity, the resistance value is mid, denoted as RGmid. The resistance values of resistors R1 and R2 are *R*_1_ and *R*_2_, and the voltages at points A and B are *V*_1_ and *V*_2_, respectively.

We adjust the resistance values of R1 and R2 so that both *V*_1_ and *V*_2_ satisfy the following conditions:

- When G receives vertically polarized light: both *V*_1_ and *V*_2_ data can be latched to 1 by the latch, i.e., recognized as high level 1;
- When G receives horizontally polarized light: *V*_1_ is latched to 1 and *V*_2_ is latched to 0 by the latch;
- When G receives no-light state: both *V*_1_ and *V*_2_ data can be latched to 0 by the latch, i.e., recognized as low level 0.

According to the latch’s recognition of high and low levels (1s and 0s), the voltage value of the high level is at least 4 volts (VCC is connected to 5 volts), and the voltage value of the low level is at most 0.8 volts. Based on the principle of voltage division, it is analyzed as follows:

(a) When G receives vertically polarized light: *V*_1_ > *V*_2_≥ 4V, the following inequality (1) holds:
$$5\frac{{R_{1}+R}_{2}}{R_{1}+R_{2}+R_{Gmin}}>5\frac{R_{2}}{R_{1}+R_{2}+R_{Gmin}}\geq 4.$$ (1)
  Therefore, the following inequality (2) holds:
$$5\frac{R_{Gmin}}{R_{1}+R_{2}+R_{Gmin}}\leq 1.$$ (2)
(b)When G receives horizontally polarized light: *V*_1_≥4V, *V*_2_≤0.8V, the following inequalities (3) and (4) hold:
$$5\frac{{R_{1}+R}_{2}}{R_{1}+R_{2}+R_{Gmid}}\geq 4,$$ (3)
$$5\frac{R_{2}}{R_{1}+R_{2}+R_{Gmid}}\leq 0.8.$$ (4)
(c) When G receives no-light state: *V*_*2*_<*V*_*1*_≤0.8V, the following inequality (5) holds:
$$5\frac{R_{2}}{R_{1}+R_{2}+R_{Gmax}}<5\frac{{R_{1}+R}_{2}}{R_{1}+R_{2}+R_{Gmax}}\leq 0.8.$$ (5)

Derived from inequality (2):

$$R_{1}+R_{2}\geq 4R_{Gmin}.$$ (6)

Derived from inequality (3):

$$R_{1}+R_{2}\geq 4R_{Gmid}.$$ (7)

Comparing inequality (7) and (6) shows that (6) holds when (7) holds.

If inequality (7) is substituted into the left side of inequality (4), we get:

$$5\frac{R_{2}}{R_{1}+R_{2}+R_{Gmid}}\;\leq 5\frac{R_{2}}{{4R}_{Gmid}+R_{Gmid}}.$$ (8)

Let inequality (8) be less than 0.8 volts, then (4) must hold:

$$5\frac{R_{2}}{{5R}_{Gmid}}\leq 0.8.$$ (9)

Derived from inequality (9):

$$R_{2}\leq 0.8R_{Gmid}.$$ (10)

Derived from inequality (6):

$$R_{1}\geq 4R_{Gmin}-R_{2}.$$ (11)

From the above deduction, it can be seen that by adjusting the resistance values of R1 and R2, the three conditions (a), (b) and (c) can be satisfied at the same time. A set of data measured randomly in the experiment are shown in Table 2:

**Table 2:** Resistance values and ranges of G, R1 and R2 (Unit: MΩ).

*R*_Gmin_	*R*_Gmid_	*R*_Gmax_	Maximum of *R*_2_	Range of *R*_1_
1.560	3.340	6.000	≤2.672	If *R*_2_ = 2.672, *R*_1_≥3.568
				If *R*_2_ = 2.500, *R*_1_≥3.740

According to the calculation, when *R*_2_ fixed a resistance value, *R*_1_ resistance value can theoretically take any value in the range of the formula (11). Considering the dispersion of the photoresistor characteristic of G and the dispersion of the output light intensity of TOP, which cause the values of R1 and R2 to deviate in each voltage divider circuit, an adjustable resistor is used as R1 in the specific implementation of the circuit shown in Fig 5, and R1 is adjusted to put the whole circuit in a good condition before the voltage division circuit is operated.

Therefore, the voltage divider circuit shown in Fig 5 can realize the three-value to two-value conversion, and the specific voltage dividing result is: whenG receives vertically polarized light, the input values of D1 and D3 are both 1 (high level) after dividing, i.e., the high voltage is converted to 11; when G receives no-light state, the input values of D1 and D3 are both 0 (low level) after dividing, i.e., the low voltage is converted to 00; when G receives horizontally polarized light, the input value of D1 is 1 (high level) after dividing, and the input value of D3 is 0 (low level), i.e., the medium voltage is converted to 10.

After the voltage divider circuit, the two binary outputs of O1 and O3 are the conversion result of one ternary electrical signal. In order to maintain consistency with the basic TOC coding, it is necessary to convert the binary data here to the representation of the TOC convention.

#### 3. Principle of BST

**Fig 6:**
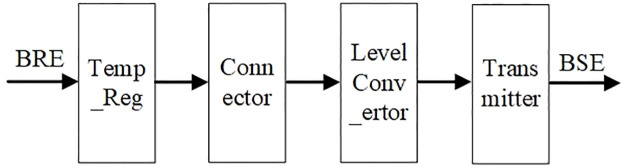
Principle of binary signal transmitter.

The role of the binary signal transmitter is to ensure that the logical format and physical state of the transmitted data are suitable for memory, with its principle shown in Fig 6. The BRE (Binary Result Electronics) signal on the left is a binary signal that conforms to the TOC convention. First, the BRE signal is temporary stored by a Temp_reg device, and then the BRE signal is spliced as required. For example, the SD16 single module is 192-processor-trit, which corresponds to 384-bit. If the data bus width of the memory is 64-bit, the 384-bit data needs to be split into six sets of 64-bit data. Next, the level of the sent data is converted to a level suitable for the memory by the Level Converter, and then passed to the Transmitter for sending. The sent signal is noted as BSE (Binary Storage Electronics).

### C. Structural design of memory data output channel

**Fig 7:**
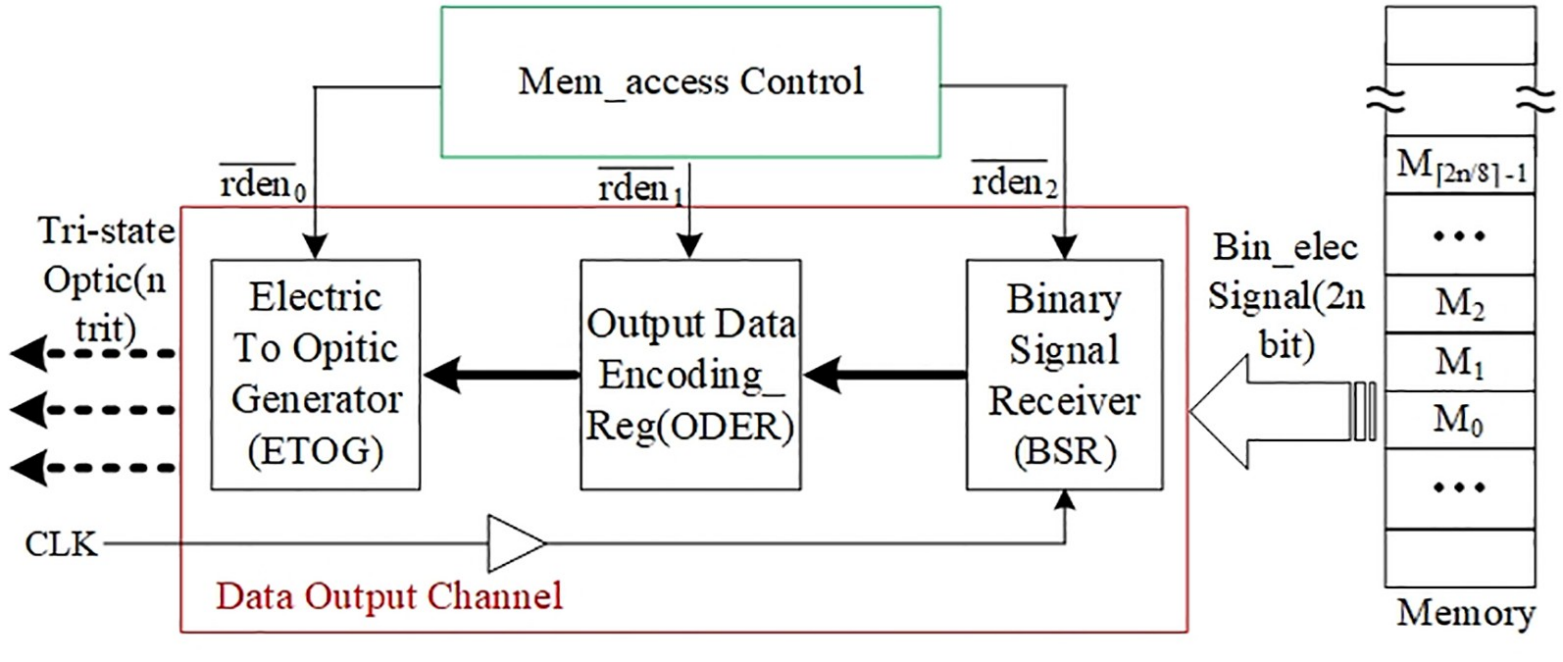
Structure of the memory data output channel.

The memory data output channel is required to accomplish the function of reading binary data from the memory and converting it into tri-state optical signal. The detailed structural design is shown in Fig 7.

The rightmost memory in Fig 7 is the same as the memory on the right side of the data input channel in Fig 3, in which 2n-bit electrical signals are stored. The Mem_access Control at the top of Fig 7 controls the modules of the data output channel in the red box to coordinate their work and complete the electro-optical conversion. The Binary Signal Receiver (BSR), under the control of signal ${\bar{rden}}_{2}$, firstly receives binary data from the specified address of the memory, and converts the physical state of the data into a level suitable for the interface through a level converter. Then, the BSR performs a separation operation on the data as needed, and sends the data to the Output Data Encoding Register (ODER) under the control of signal ${\bar{rden}}_{1}$. Since the data stored in the memory is a basic encoding of two binary data expressing one ternary data, no further encoding conversion is required for the output channel.

**Fig 8:**
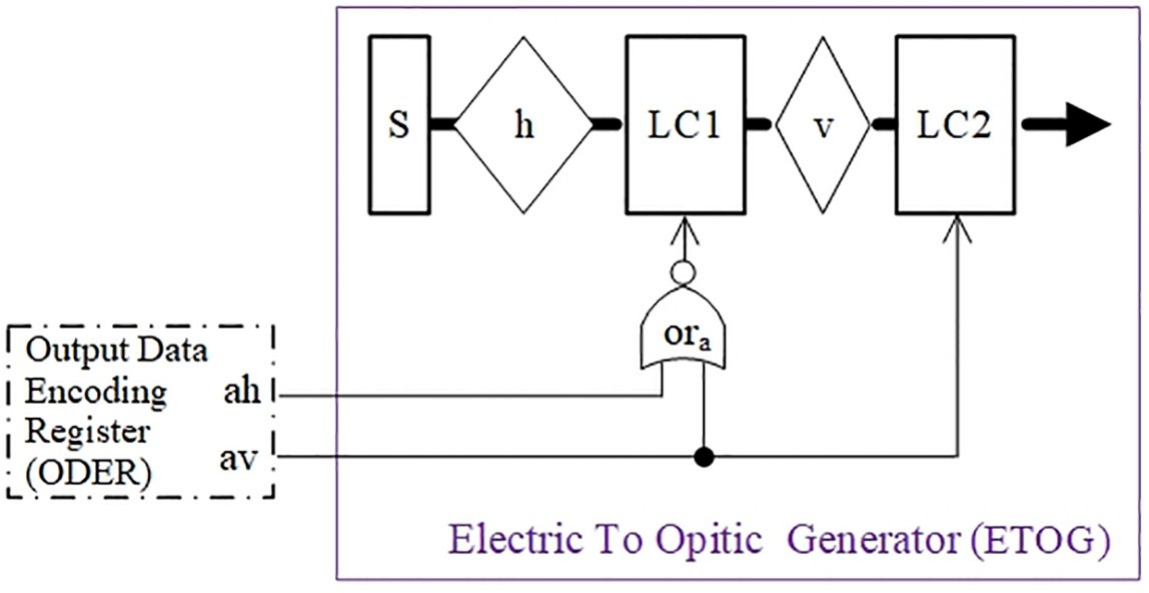
Schematic diagram of optical signal generator.

The role of Electric To Opitic Generator (ETOG) is to convert a binary signal into a tri-state optical signal, and Fig 8 demonstrates the principle of generating one tri-state optical signal.

The specific conversion process is as follows: firstly, the 2n-bit signal is separated into n groups of binary data with high bits (noted as ah) and low bits (noted as av), where ah and av are stored separately in the ODER. Then, ah and av are fed into the ETOG, respectively. The ETOG adopts the structure of two layers of polarizer sandwiching two layers of liquid crystal in Fig 8. The first layer being a horizontally polarized sheet h, and the natural light emitted from the light source S passes through the sheet h and forms horizontally polarized light. The second layer is the liquid crystal LC1; the third layer is the vertically polarized sheet v; and the fourth layer is the liquid crystal LC2. Based on the aforementioned TOC convention expression, (ah, av) takes three values: (1, 0) for horizontally polarized light state, (0, 1) for vertically polarized light state, and (0, 0) for no-light state.

The NOR operation result of ah and av is connected to liquid crystal LC1, for controlling LC1 optical rotation. Assuming that the liquid crystal with normal optical rotation is selected, when (ah, av) takes (0, 0) for the representation of no-light state, LC1 does not rotate the light (change the polarization direction). So the light arrives at the vertical polarizer v is still a horizontally polarized light, which can not pass through the polarizer v, and then there is no light to reach LC2. As a result, there is no light emitted from the ETOG, that is, the binary electrical signals (0, 0) is converted into a tri-state optical signal of no-light state.

When the vertically polarized light state enters the ETOG, (ah, av) takes (0, 1). Then, the horizontally polarized light is rotated into vertically polarized light by LC1, and can pass through the vertical polarizer v. The signal av controls LC2 making it not rotate the light, so ETOG produces vertically polarized light, that is, the binary electrical signal (0, 1) is converted into a tri-state optical signal of vertically polarized light.

When the horizontally polarized light state (1,0) enters the ETOG, (ah, av) takes (1, 0). Then, the horizontally polarized light is rotated into vertically polarized light by LC1, and can pass through the vertical polarizer v. The signal av controls LC2 making it rotate the light. So, the vertically polarized light is rotated into horizontally polarized light by LC2. As a result, ETOG generates horizontally polarized light, which converts the binary electrical signal (1, 0) into a tri-state optical signal of horizontally polarized light.

The optical signal generator described above converts the 2-bit signals (ah, av) to one of the three optical signals, namely no-light state, vertically polarized light state, and horizontally polarized light state, so that the 2-bit signals are converted to 1-trit optical signal. Constructing n ETOGs as shown in Fig 8 completes the conversion of the 2n-bit signals to n-trit optical signals.

## 4 The working process of memory interface

### A. Read and write timing of the memory interface

**Fig 9:**
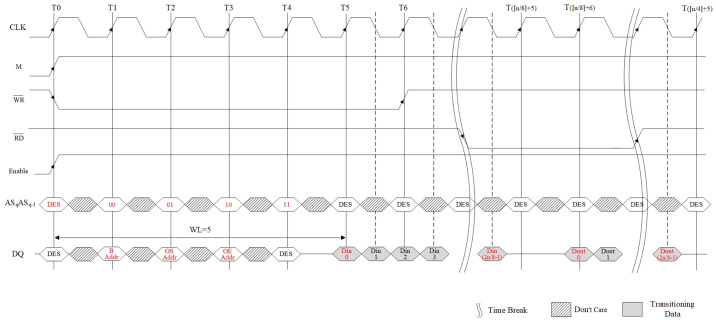
Memory interface read/write timing diagram.

According to the above design principle and structure of the memory interface, its read and write timing is shown in Fig 9.

The write timing of the data input channel is as follows: at the moment T0 on the rising edge of the clock, the control signal M goes from low to high; the memory access takes effect; the write signal $\bar{WR}$ and the interface enable signal (Enable) also take effect synchronously. At this time, the clock and control signals are in an unstable state. In order to ensure that the data are correct, the data on the address and data buses are not sampled at this moment, corresponding to the "DES (Deselect)" in Fig 9. After a clock cycle, the control signals have been stabilized. So, at time T1, the highest two-bit data ASqASq-1 on the address bus are collected. Firstly, the data 00 are collected, when the BAR receives the address data B Addr sent on the data bus. Then, at time T2, the data 01 are collected, when the offset address start register (OASR) receives the OS Addr. At time T3, the data 10 are collected, when the offset address end register (OAER) receives the OE Addr. Finally, at time T4, the data 11 are collected, when the interface controller is ready to start controlling write operation of the data. At time T5, the first input data Din0 on data bus is collected, that is, the Write Latency (WL) is 5 cycles. In order to speed up data transmission, data are also received on the falling edge of the clock. Until the time T⌈n/8⌉+5, the data writing is completed.

The read timing for data output channel is similar to the write timing. Fig 9 also shows the timing of writing followed by reading for the same memory address. At the T⌈n/8⌉+1 moment on the rising edge of the clock, the read signal $\bar{RD}$ starts to take effect from high to low. Similar to the writing process, the Read Latency (RL) and WL are the same as 5 clock cycles, and the first output data Dout0 is captured at the time T⌈n/8⌉+6. Until the time T⌈n/4⌉+5, the data reading is completed.

### B. Working process of the memory interface

**Fig 10:**
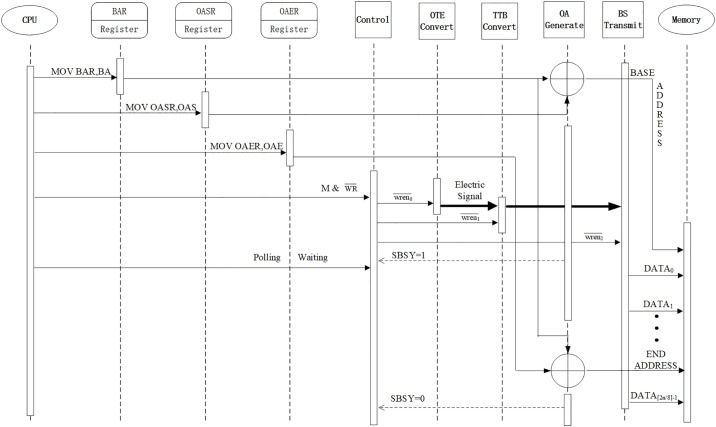
Working process of input channel.

According to the write timing in Fig 9, the working process of the input channel is shown in Fig 10. The left and right sides of Fig10 are the CPU and memory, respectively. The center is the read/write controller. On the left side of the read/write controller are three address registers, namely, BAR, OASR and OAER; on the right side of the interface controller are four devices, namely, photoelectric converter, ternary to binary converter, offset address generator, and binary signal transmitter. At the first step, CPU sends the base address represented by BA into the register BAR via the instruction "MOV BAR, BA". At the second step, CPU sends the starting offset address represented by OAS into the register OASR via the instruction "MOV OASR, OAS". At the third step, CPU sends the ending offset address represented by OAE into the register OAER via the instruction "MOV OAER, OAE". At the fourth step, CPU starts the read/write controller by outputting a high level to the M signal that accesses the memory and a low level to the write signal $\bar{WR}$, via the write memory instruction. The read/write controller then sends a ${\bar{wren}}_{0}$ signal to the photoelectric converter to initiate the photoelectric conversion process to convert the tri-state optical signal to an electrical signal. Next, the read/write controller sends a ${\bar{wren}}_{1}$ signal to TTBC to initiate the conversion process to convert ternary electrical signals into binary electrical signals according to the TOC convention. After that, the read/write controller sends a ${\bar{wren}}_{2}$ signal to the binary signal transmitter to initiate the data transmission process. The offset address generator sets the busy status signal SBSY to 1 and returns it to the read/write controller. After the binary signal transmitter sends the first set of data Data0, it sends subsequent data (Data1, Data2, …, Data2n-1) in turn until the last data Data2n-1, according to the address automatically generated by the offset address generator. When the last set of data is sent, the offset address generator sets the busy status signal SBSY to 0 and returns it to the read/write controller. During the process of writing data to the memory, CPU uses polling to wait for the completion of writing data.

The working process of the output channels is similar and will not be discussed again.

## 5 Validation experiments and analysis

**Fig 11:**
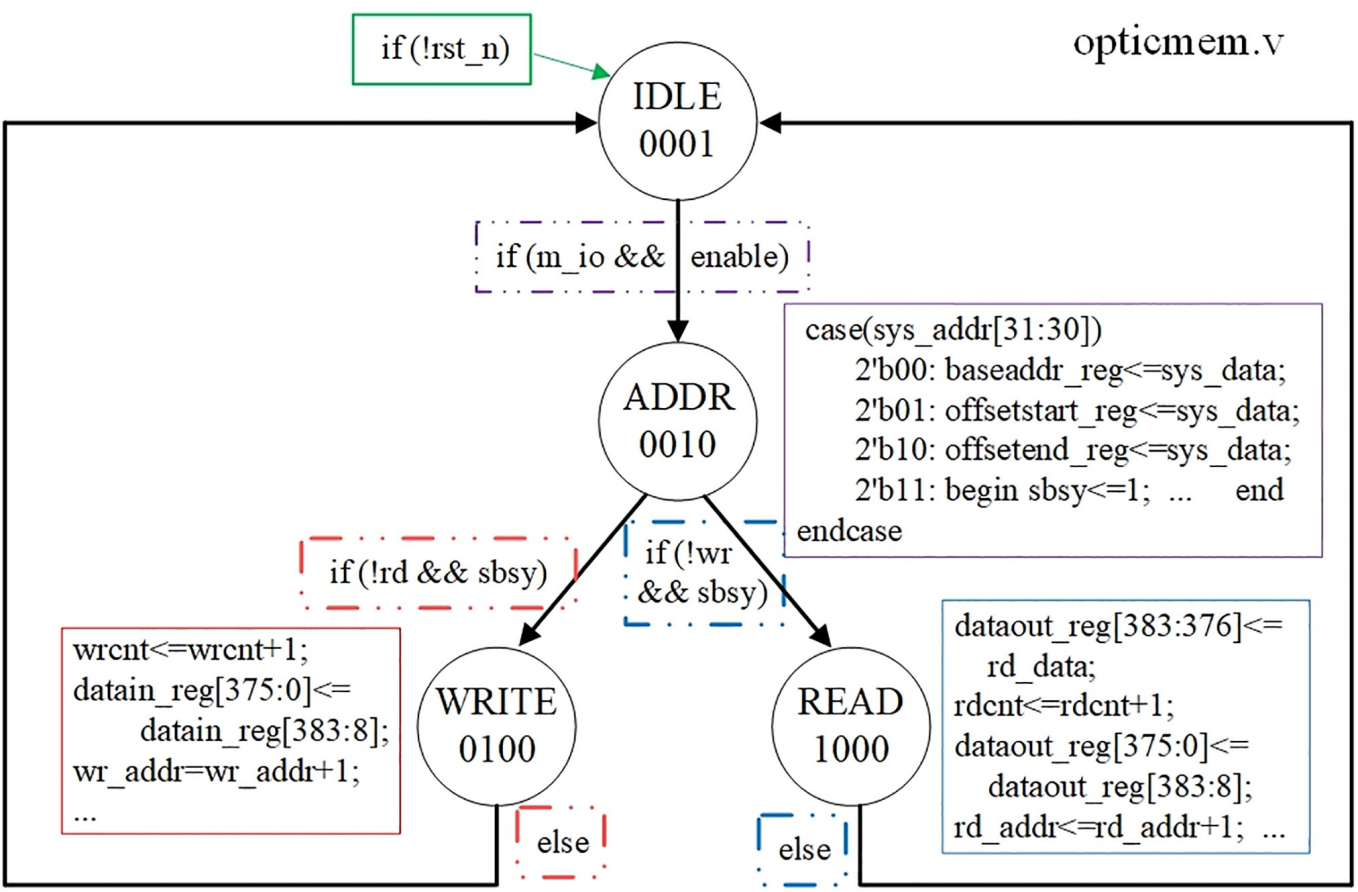
State transition diagram of experimental design.

We use FPGA to verify the correctness of the structural design of TOC memory system The state transition diagram of the experimental design is shown in Fig 11.

In the experiment, there are four states, IDLE, ADDR, WRITE and READ, with corresponding codes of 0001, 0010, 0100, and 1000, respectively, as indicated by the four circles in Fig 11. The IDEL state is entered when the memory system receives the reset signal rst_n, and the entry conditions are shown in the green box at the top of Fig 11. At the ADDR state, the addresses from the system bus, including the base address baseaddr_reg, the starting offset address offsetstart_reg, and the ending offset address offsetend_reg, are stored into the three registers BAR, OASR and OAER, respectively. The purple solid line box in Fig 11 illustrates the functional design of the 2–4 decoder in Fig 2. The condition for entering the ADDR state is that the memory access signal M and the 2–4 decoder enable signal are both high, as indicated by the purple dotted line box in Fig 11. At the WRITE state, the binary data generated by the data input channel (Fig 3) are written into the memory, where the red solid line box in Fig 11 demonstrates its main function. The condition for entering the WRITE state is that the write signal *wr* is low and the memory busy signal *sbsy* is high, as indicated by the red dotted box in Fig 11. At the READ state, the data read from the memory are sent to the aforementioned data output channel, where the blue solid line box in Fig 11 demonstrates its main function. The condition for entering the READ state is that the read signal *rd* is low and the memory busy signal *sbsy* is high, as indicated by the blue dotted box in Fig 11. As with the WRITE state, when the entry condition is not met (“else” in the two dotted boxes at the bottom of Fig 11), it jumps back to the IDLE state.

**Fig 12:**
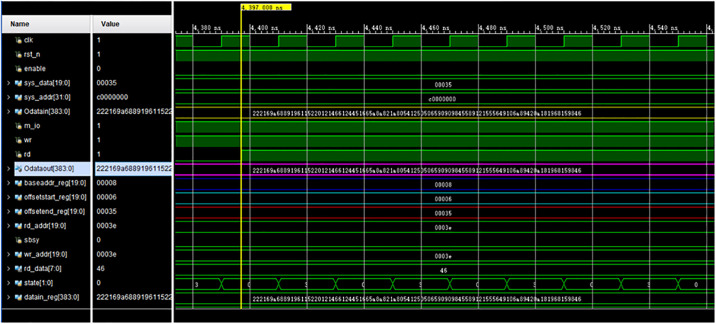
FPGA experimental simulation results.

The experiment was carried out on the operand data of a single module of the TOC prototype SD16. The data are 192-trit ternary data, so that the value of n is 192 in this experiment, which is converted to 2n, i.e., 384-bit data, by the data input channel. The goal of the experiment is to write the 384-bit data to the memory and read them out correctly to the output channel. The simulation results in Fig 12 show a comparison of the writing and reading results of one set of data 222169a6889196115220121466124451665a8a821a80541250506590909845589121555649106a89420a181968159846, which are 384-bit data in hexadecimal. It can be seen that when the read signal rd becomes high level (the position marked by the yellow vertical line in Fig 12), the read data marked in purple and the write data marked in yellow are exactly the same, which verifies the correctness of the aforementioned memory system design.

We carried out repeated experiments, and the data reading and writing results were correct. Table 3 below shows 10 groups of experimental test cases.

**Table 3:** 10 groups of experimental test cases.

No.	384-bit test cases represented in hexadecimal form	R/W test result
1	1417e57e61b7f386297bc85677a2b07e8ff355abe0a5c10d230fda72df908ed5c59 039b6fe70ff66df83c4d8b20e3667	√
2	c2420d892c55cf5577829613693fefd0ca66b3e9e125ed2601e303d802c25a5dc9 aa2971988405add13bbb613f49c35a	√
3	3175ac5acd90bb8efbef41149d071187a082b32c1421d95b209ae3ec37eb757bad dc0ad50c1d933932a1547cd1e6e8f8	√
4	e04cf8ce2e2e0b15fbf5e222422ca15f62655bd736fc0bd2b943a5acf43a8df8b43 a8502eca988b5ca29b80a6a882b84	√
5	db35111308c8b5b97762fa838e7e24844302ffe7ba9e3ce536f5af59fb46f3c6636 24123e4dbb191aaacc2a7a0ea4c40	√
6	d28fd12153bdecc229c38c60edb0a657d8a0a8e397aa9320b60f7c2ac76a634f55 35ffe0390485f5721f360078a715f8	√
7	e2820c3573302499961929db3d34522ff704d8390a5790aaf5d7425fbaa1c7807e f54218d1260f139b37a710c0839c0c	√
8	0b279cf92451aea7260d9a87af881ea1628e1251bda21a92ef8f213b782adcac9c de2baa31fe07de9585862054c718af	√
9	9bc8adece8c456d9c68615f2ce2ebef00db9af42cc170c6974d48101947d3d4ba 51ab25db96802146b2c1035ea8833db	√
10	1c6b8ccc02bb3f9fdd221f1940d0be0d1f1291874f07273c3330dd5dea7b68e03 50d198babc37378a899c663208f4eeb	√

## 6 Summary

This paper discusses the design and implementation of the interface structure based on TOC memory system. After the discussion of the characteristics and requirements of the TOC memory interface, the overall structure of the memory interface is constructed, and then the design principle and structure of the input channel module, the working process of the input channel, and the structural design of the output channel are elaborated in detail. Finally, the validity of the interface design is verified through the experiment of the TOC operand data on FPGA. This lays a solid foundation for the further development of TOC.

With the further development of TOC application [20, 21], we will need larger capacity, lower power consumption and NVRAM memory. Low-power SRAM [22, 23] and NVRAM [24] provide us with useful references,, which is one of the research directions for the future.
